# Enhanced therapeutic effect using sequential administration of antigenically distinct oncolytic viruses expressing oncostatin M in a Syrian hamster orthotopic pancreatic cancer model

**DOI:** 10.1186/s12943-015-0479-x

**Published:** 2015-12-16

**Authors:** Estanislao Nistal-Villan, Maria Bunuales, Joanna Poutou, Manuela Gonzalez-Aparicio, Carlos Bravo-Perez, Jose I. Quetglas, Beatriz Carte, Gloria Gonzalez-Aseguinolaza, Jesus Prieto, Esther Larrea, Ruben Hernandez-Alcoceba

**Affiliations:** Gene Therapy Program. CIMA Foundation for Applied Medical Research, University of Navarra, Pamplona, Spain; IdiSNA, Navarra health research institute, Pamplona, Spain; CIBERehd, University Clinic of Navarra, Pamplona, Spain; Instituto de Salud Tropical, University of Navarra, Pamplona, Spain

**Keywords:** Oncolytic viruses, Pancreatic cancer, OSM, Adenovirus, NDV

## Abstract

**Background:**

The limited efficacy of current treatments against pancreatic cancer has prompted the search of new alternatives such as virotherapy. Activation of the immune response against cancer cells is emerging as one of the main mechanisms of action of oncolytic viruses (OV). Direct oncolysis releases tumor antigens, and viral replication within the tumor microenvironment is a potent danger signal. Arming OV with immunostimulatory transgenes further enhances their therapeutic effect. However, standard virotherapy protocols do not take full advantage of OV as cancer vaccines because repeated viral administrations may polarize immune responses against strong viral antigens, and the rapid onset of neutralizing antibodies limits the efficacy of redosing. An alternative paradigm based on sequential combination of antigenically distinct OV has been recently proposed.

**Methods:**

We have developed a protocol consisting of sequential intratumor administrations of new Adenovirus (Ad) and Newcastle Disease Virus (NDV)-based OV encoding the immunostimulatory cytokine oncostatin M (OSM). Transgene expression, toxicity and antitumor effect were evaluated using an aggressive orthotopic pancreatic cancer model in Syrian hamsters, which are sensitive to OSM and permissive for replication of both OVs.

**Results:**

NDV-OSM was more cytolytic, whereas Ad-OSM caused higher OSM expression in vivo. Both viruses achieved only a marginal antitumor effect in monotherapy. In addition, strong secretion of OSM in serum limited the maximal tolerated dose of Ad-OSM. In contrast, moderate doses of Ad-OSM followed one week later by NDV-OSM were safe, showed a significant antitumor effect and stimulated immune responses against cancer cells. Similar efficacy was observed when the order of virus administrations was reversed.

**Conclusion:**

Sequential administration of oncolytic Ad and NDV encoding OSM is a promising approach against pancreatic cancer.

**Electronic supplementary material:**

The online version of this article (doi:10.1186/s12943-015-0479-x) contains supplementary material, which is available to authorized users.

## Background

Pancreatic cancer is one of the most aggressive human solid tumors. Typical characteristics include rapid growth, infiltration and early metastatic spread. Surgical resection with negative margins continues to be the only potential cure, but only 10–20 % of patients with pancreatic ductal adenocarcinoma (PDAC) have primarily resectable disease at first diagnosis. Forty percent will have metastatic disease, and another 30–40 % will have locally advanced neoplasms. This results in a median overall survival in patients with primarily resectable tumors of 20–24 months, and 9–13 months in locally advanced tumors, with a 95 % mortality rate five years after diagnosis [1, 2]. Thus, there is an urgent need to identify effective therapies for pancreatic cancer.

Oncolytic viruses (OV) adapted as gene therapy vectors for the expression of immunostimulatory cytokines are emerging as a promising approach for the treatment of solid tumors [3]. To date, the most prominent and clinically advanced representatives of this new class of agents are the herpes simplex virus type 1-derived T-VEC and the vaccinia virus-derived Pexa-Vec viruses, both expressing granulocyte-macrophage colony-stimulating factor (GM-CSF) as a therapeutic gene [4]. Recent trials provide proof of concept for clinical efficacy in advanced melanoma (T-VEC) [4, 5] and hepatocarcinoma (Pexa-Vec) [6], beyond that observed with previous versions of these OVs lacking therapeutic genes. The proposed mechanism of action includes the combination of viral effects (direct oncolysis, release of tumor antigens, danger signals and inflammation) and the additional stimulation of the immune system mediated by the cytokine expressed from the virus. Since tolerance to these agents has been generally good, intensification of treatments using higher doses or more potent cytokines would be the next logical step to increase response rates. However, antiviral neutralizing antibodies induced after the first virus dose may inhibit the infectivity of subsequent vector administrations. Furthermore, repeated vector doses may stimulate cellular immune responses against the virus that might outcompete antitumor responses. It should also be considered that increasing viral doses to compensate for a reduction in the number of administrations is not always possible, particularly when high transgene levels cause toxicity.

An alternative to these protocols is the sequential administration of antigenically distinct viruses. The principle of prime and boost is employed for vaccination against different pathogens [7], and it has been recently proposed in the field of OV with the objective of increasing the immune responses against tumor antigens. Tysome et al. demonstrated that sequential intratumoral administration of wild type adenovirus (Ad) followed by vaccinia virus has a greater therapeutic effect than repeated doses of the same virus, and that stimulation of cellular immune responses against cancer cells plays an important role [8]. We hypothesize that this concept can be applied to other OV combinations and that the incorporation of potent immunostimulatory cytokines as therapeutic genes in these viruses can further enhance their antitumor effect. The use of different armed viruses without cross humoral responses would allow at least two short cycles of transgene expression. The duration of each cycle will be intrinsically limited by the tumor cells, the viral replication cycle and the cytolytic nature of the viruses. Therefore, we consider that cytokines that promote antigen presentation and co-stimulatory signals such as oncostatin M (OSM) are highly suited to arm OV aimed at triggering antitumor immunity. OSM belongs to the interleukin (IL)-6 family, together with IL-6, cardiotrophin-1, IL-11, leukemia inhibitory factor (LIF), and ciliary neurotrophic factor [9]. OSM binds at least two heterodimeric transmembrane receptors on the surface of cells, one composed of the specific OSM receptor subunit (OSMR) plus the gp130 moiety, and another comprising gp130 plus the LIF receptor (LIFR) subunit [10]. The receptors are expressed in connective tissues, neurons and a variety of epithelial cells. Downstream signaling involves Janus tyrosine kinases (Jak1, Jak2 and Tyk2) as well as STAT1 and STAT3 transcription factors [11]. OSM exerts different biological effects depending on the pattern and localization of expression. It can be secreted by monocytes, macrophages, dendritic cells and neutrophils as a mediator of acute inflammatory responses, similar to IL-6 [12–16]. In addition, OSM acting on hepatocytes plays a role in the adaptive response against viral hepatitis by stimulating the expression of genes involved in activation and expansion of lymphocytes (such as IL-15Rα and ICAM1) [17, 18]. When OSM acts in the presence of type I interferon (IFN), it can also activate the machinery of antigen processing and presentation in epithelial cells by increasing the expression of HLA, β2-microglobulin, Tap1, Tap2 and proteasome subunits, among others [17]. Short-term treatment of hepatocellular carcinoma with recombinant OSM promoted cell differentiation and increased susceptibility to chemotherapy in vitro and in vivo [19]. Similarly, transient expression of OSM using gene therapy vectors has shown antitumoral effect in melanoma models, in part through inhibition of cell proliferation and induction of apoptosis [20]. In contrast, chronic OSM production has been associated with progression of other tumors such as breast cancer, through stimulation of angiogenesis, epithelial-mesenchymal transition and invasiveness [21]. Therefore, the functional profile of OSM is compatible with its use as a therapeutic gene in the context of transient expression by OVs.

In the present study, we produced oncolytic Ad and Newcastle disease virus (NDV) carrying this transgene. Ad is a double-stranded DNA virus with naked capsids of approximately 100 nm in diameter. It presents moderate oncolytic potency and is very efficient in priming immune responses [22]. In addition Ad has a very stable non-integrating genome and efficient recombinant gene expression machinery. Ad can be routinely produced to high titers for clinical use and research has long confirmed the safety of these agents as OV in patients [23]. NDV is a single negative stranded RNA paramyxovirus with membrane-enveloped particles of 150–350 nm in diameter [24]. Its lytic cycle is faster than Ad (24 h versus 48 h, on average), and it favors a pro-inflammatory microenvironment in tumors, through stimulation of type I IFN and chemokine production [25], as well as activation of macrophages [26] and natural killer cells [27], which modify the local tumor microenvironment and generate a potent local antitumor response. Few individuals present neutralizing NDV antibodies and it is one of the safest OV for humans since it is a bird adapted virus with little chances of adapting to the human host. Furthermore, it has tumor selective replication properties [24, 28].

In the present study, we used an orthotopic pancreatic cancer model established in Syrian hamsters to evaluate the toxicity and antitumor effect of Ad and NDV expressing human OSM (hOSM), alone or in different combinations. Although tools to study hamster immune response are limited, this animal model offers important features for the study of virotherapy because it is immune competent, permissive for Ad and NDV, and sensitive to a variety of human cytokines such as OSM and IL-12 [29, 30]. In addition, hamster pancreatic cancer cell lines such as HaP-T1 recapitulate important genetic abnormalities found in human PDAC, such as the activating G12D mutation in the K-ras oncogene [31]. These characteristics, together with the aggressiveness conferred by the anatomical localization of tumors, make this model a stringent test for the safety and efficacy of armed OVs. Herein we describe the properties and limitations of each virus and highlight the importance of combination protocols to obtain the optimal therapeutic effect.

## Results

### Oncolytic Ad and NDV replicate and destroy pancreatic cancer cells from humans and Syrian hamsters

**Fig. 1:**
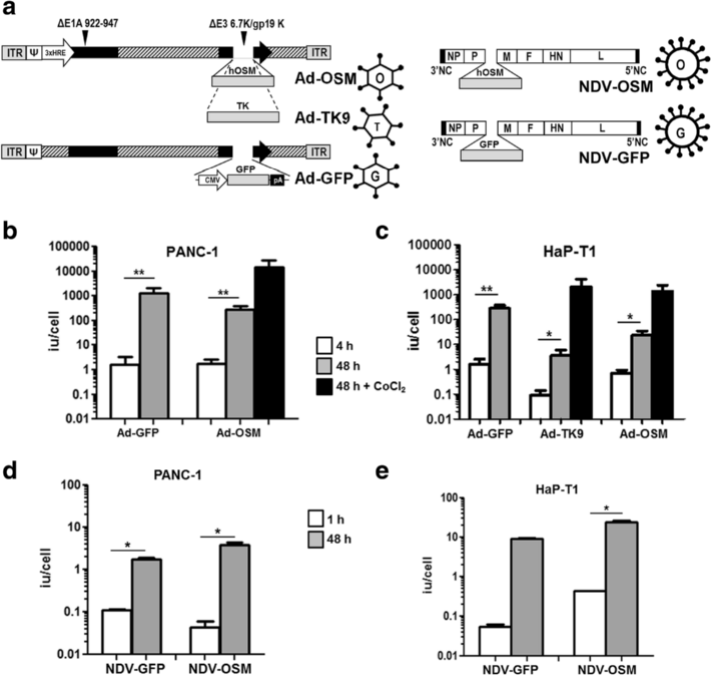
Ad-OSM and NDV-OSM replicate in human and hamster PDAC cells. **a** Schematic representation of the main viruses used in this study. ITR, adenovirus inverted terminal repeat; ΔE1A 922–947 indicates the nucleotides deleted from the E1A gene; ΔE3 6.7/gp19K indicates the deletion of these genes in the E3 region; GFP, enhanced green fluorescence protein; TK, thymidine kinase from HSV-1; NP, P, M, F, HN and L are NDV genes. **b**-**e** Quantification of infectious particles produced at the indicated times in PANC-1 and HaP-T1 cells infected with the different viruses (representative results of at least 2 experiments performed in triplicate). Black columns correspond to cells treated with the hypoxia-mimetic drug CoCl_2._ * *p* <0.05, ** *p* <0.01

**Fig. 2:**
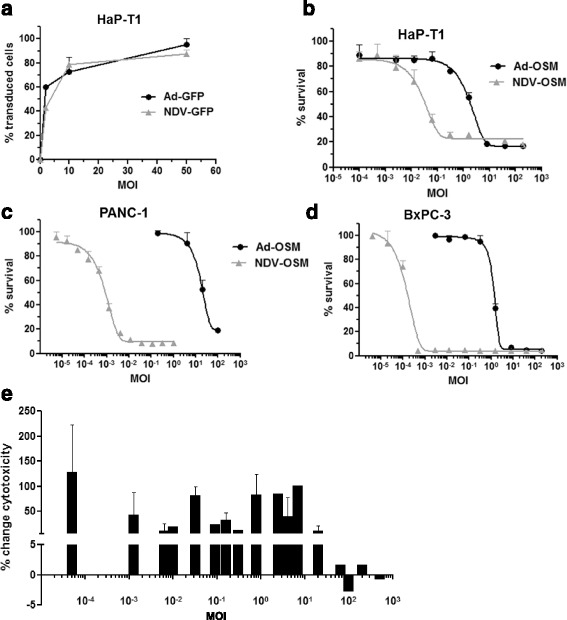
NDV-OSM shows stronger oncolytic effect than Ad-OSM on PDAC cells in vitro. **a** Percentage of transduced (GFP^+^) HaP-T1 cells 24 h after infection with Ad-GFP and NDV-GFP at the indicated MOIs. **b-d** cells were infected at different MOIs (X-axis, logarithmic scale) and the percentage of viable cells remaining in the monolayer was quantified 5 days later by crystal violet staining (representative results of at least 2 experiments with 4 samples per point). Uninfected cells were used as a reference (100 % survival). **e** HaP-T1 cells were co-infected with equal MOIs of Ad-OSM and NDV-OSM, or infected with NDV-OSM only. The change in cytotoxic effect (enhancement or inhibition) is represented as a positive or negative percentage, respectively, considering 0 the value obtained by single NDV-OSM infection at each MOI

The armed OVs, OAV-H-OSM and NDV-LaSota-F3aa-OSM were obtained by incorporating the hOSM cDNA sequence into the genome of oncolytic Ad and NDV viruses, respectively. Ad were derived from human serotype 5. These agents are called hereinafter Ad-OSM and NDV-OSM. In the Ad-OSM virus the transgene was placed in the E3 region, as depicted in Fig. 1a. Conditional replication of this virus was achieved by eliminating the pRB-binding domain of E1A, plus transcriptional control of E1A using a hypoxia-responsive promoter, as previously described [29, 32]. NDV-OSM carries OSM inserted between the P and M genes, and a modified fusogenic protein (F) cleavage site designed to increase its lytic and immunostimulatory properties [33] (Fig. 1a). PDAC cell lines from humans (PANC-1) and Syrian hamsters (HaP-T1) were infected with these viruses, and their amplification was determined by comparing the amount of infectious particles present in cell lysates at early times after infection (4 h for Ad-OSM and 1 h for NDV-OSM), and 48 h later. As shown in Figs. 1b-e, both human and hamster cell lines were permissive for replication of the viruses. In agreement with their respective lytic cycles, Ad-OSM produced more infectious units (iu)/cell compared with NDV-OSM. The full replicative potential of Ad-OSM was achieved when cells were incubated with the hypoxia-mimicking agent CoCl_2_, which increases the production of hypoxia-inducible factor (HIF-1α) and stimulates the hypoxia-responsive elements (HRE) present in the artificial promoter used to control E1A expression in this virus [32]. To verify that the incorporation of OSM into the genome of the OVs did not interfere with replication, we performed a side by side comparison with replication-competent viruses expressing the reporter gene eGFP or the suicide gene thymidine kinase (TK) from HSV-1 (Fig. 1a). NDV-LaSota-F3aa-EGFP (NDV-GFP) incorporates eGFP between the P and M genes. Ad-TK9 is identical to Ad-OSM except for the presence of the TK gene instead of hOSM. Ad-WT-CMV-GFP (referred to here as Ad-GFP) is identical to the wild type human Ad5, except for the incorporation of an expression cassette for eGFP in the E3 region. Therefore, it presents unrestricted replication. No significant differences were observed in the amplification of viruses containing OSM and GFP or TK in any of the cell lines (Figs. 1b-e). Next, we studied the oncolytic (cytopathic) effect of these viruses in vitro, and confirmed that the incorporation of OSM does not alter their potency (data not shown). Interestingly, NDV showed a stronger cytopathic effect in HaP-T1 cells compared with Ad (Fig. 2b), despite equivalent infectivity (Fig. 2a) and a lower amplification index (Fig. 1c,e), reflecting the differences in the lytic cycle of both viruses. The enhanced oncolytic potency of NDV-OSM was particularly evident in human PDAC cells, PANC-1 and BxPC-3 (Fig. 2c,d), despite equivalent infectivity (data not shown). To rule out the possibility that Ad and NDV could inhibit each other in case of co-infection, we studied the cytotoxic effect obtained in HaP-T1 cell cultures co-infected with Ad-OSM and NDV-OSM, in comparison with single NDV-OSM infection. In Fig. 2e we represent the change in cytotoxicity of NDV-OSM caused by the addition of Ad-OSM at each MOI. Our results indicate that there is virtually no change in the potency of NDV-OSM at high MOIs, whereas a moderate increase is observed at low-intermediate MOIs. This result indicates that Ad does not interfere with the oncolytic effect of NDV.

### Human and Syrian hamster PDAC cells respond to recombinant or virally encoded hOSM

**Fig. 3:**
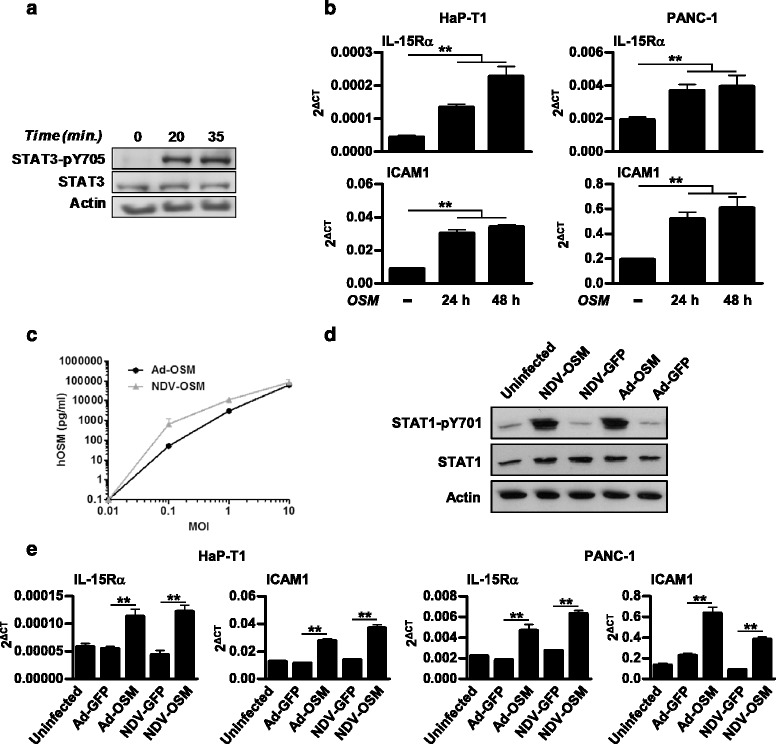
Human OSM is biologically active on PANC-1 and HaP-T1 cell lines. **a** Western blot analysis of STAT3 activation in HaP-T1 cells treated with 20 ng/ml of recombinant hOSM at the indicated times. **b** mRNA levels of IL-15Rα and ICAM1, determined by qRT-PCR in HaP-T1 and PANC-1 cell lines treated with 20 ng/ml of recombinant hOSM at the indicated times. **c** Concentration of hOSM in the conditioned medium of HaP-T1 cells infected for 24 h with the indicated MOIs of Ad-OSM and NDV-OSM, determined by ELISA. **d** Western blot analysis of STAT1 activation in HuH-7 cells treated with conditioned medium from HaP-T1 cells infected or not with Ad-OSM, NDV-OSM or their respective GFP-expressing controls. **e** mRNA levels of IL-15Rα and ICAM1, determined by qRT-PCR, in HaP-T1 and PANC-1 cell lines treated for 24 h with the same conditioned media (representative results of at least 2 experiments performed in triplicate). ** *p* <0.01

To investigate the biological activity of hOSM in Syrian hamsters, HaP-T1 cells were incubated with 20 ng/ml recombinant hOSM, and phosphorylation of STAT3 was detected by western blot analysis at different times as an indicator of downstream signaling. As shown in Fig. 3a, a rapid activation of STAT3 was detected in these cells. Furthermore, hOSM treatment caused up regulation of downstream genes such as IL-15Rα and ICAM1 with similar intensity in human and Syrian hamster PDAC cell lines, as shown in Fig. 3b. Next, HaP-T1 cells were infected with Ad-OSM, NDV-OSM or their reporter virus counterparts, and conditioned medium was collected 48 h later for quantification of hOSM by ELISA. As shown in Fig. 3c, the cytokine was detected in cells infected with both hOSM-expressing viruses. The biological activity of the virally encoded hOSM was verified by incubation of the liver-derived human cell line HuH-7 with these media. As previously reported [17], hOSM stimulated the phosphorylation of STAT1 in these cells (Fig. 3d). Of note, no type I IFN activity was detected in these conditioned media from HaP-T1 cells by bio-assay (data not shown), and by analysis of IFN-stimulated genes mRNA expression on hamster cell lines (Additional file 1: Figure S1). These results indicate that Ad-OSM and NDV-OSM achieve expression of functional hOSM in infected cells, and neither of these viruses stimulates production of type I IFN in vitro in this particular cell line. In agreement with this observation, HaP-T1 and PANC-1 cells directly infected with Ad-OSM or NDV-OSM showed specific stimulation of OSM downstream genes such as IL-15Rα and ICAM1 (Fig. 3e), but not others associated with the combination of OSM and IFNα such as Tap1 or β2-microglobulin (data not shown). Together, these in vitro results indicate that infection of PDAC cells with Ad-OSM or NDV-OSM viruses achieves secretion of functional hOSM, which is active in both human and hamster cells and stimulates the expression of immunostimulatory genes.

### Toxicity and antitumor effect of Ad-OSM and NDV-OSM in vivo

**Fig. 4:**
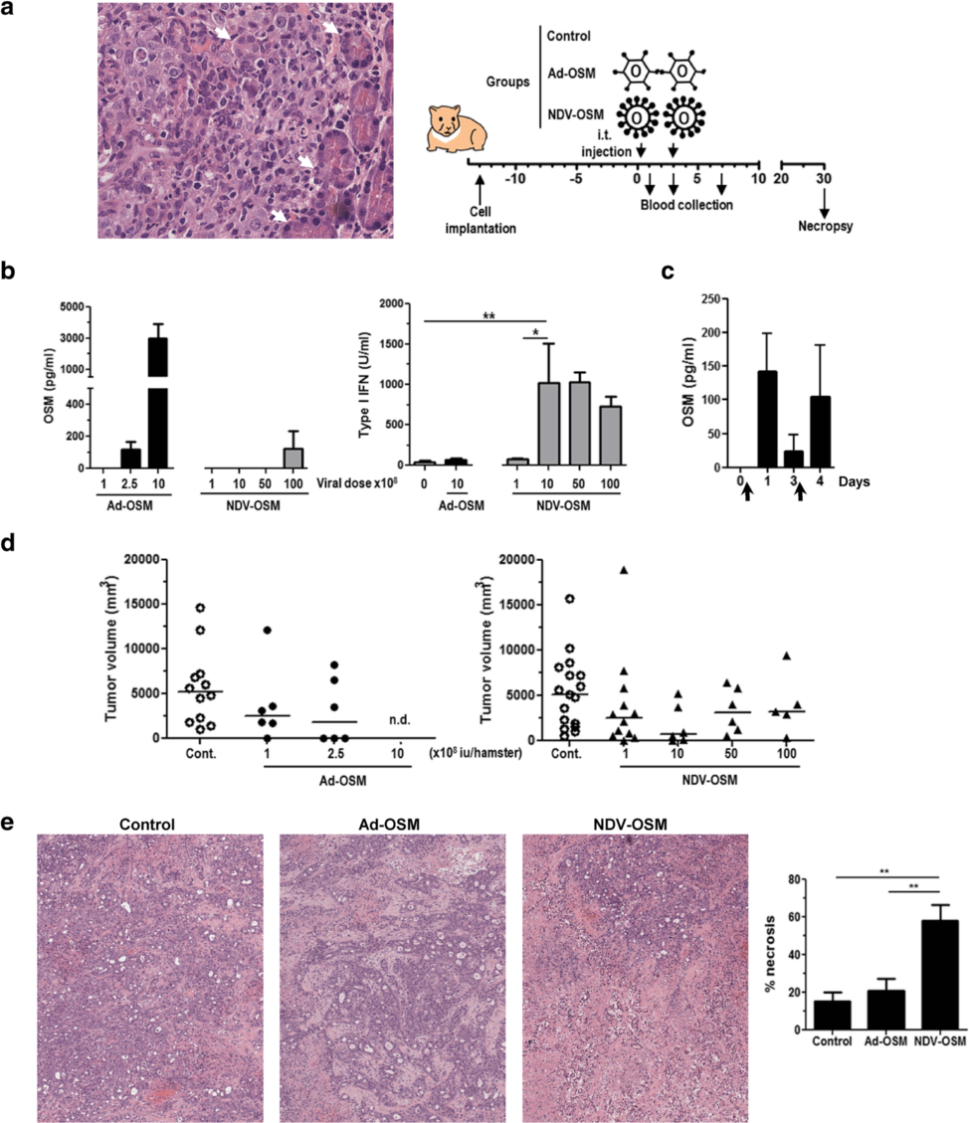
Ad-OSM and NDV-OSM show limited antitumor effect as monotherapy. Tumors were established by intrapancreatic inoculation of HaP-T1 cells. **a** Left panel shows the histopathological examination of a representative tumor (Hematoxylin/Eosin staining, magnification x400). Note the infiltration of normal pancreas and engulfment of secretory acini (white arrows). Right panel shows a schematic representation of the experimental setting. Treatments started 2 weeks after cell implantation (referred to as day 0) by intratumoral (i.t.) injection of Ad-OSM or NDV-OSM (*n* = 6-12). Control animals received the same volume of saline solution (*n* = 12-16). A second dose of each virus or saline was administered at day 3. All hamsters were sacrificed one month after the initiation of treatment. **b** Concentration of hOSM and type I IFN in the serum of hamsters 24 h after treatment with the indicated viruses, determined by ELISA and bioassay, respectively. Viral doses correspond to i.u. (x10^8^). **c** Apart from the efficacy study, an independent group of tumor-bearing hamsters (*n* = 5) received Ad-OSM at 2.5 × 10^8^ i.u. at days 1 and 3 (arrows), and serum hOSM concentration was quantified right before and 24 h after each virus administration. **d** Individual tumor volumes and group averages determined by necropsy one month after initiation of treatments in the efficacy study. n.d., not determined due to early death of animals. **e** Additional groups of hamsters (*n* = 5) received Ad-OSM (2.5 × 10^8^ i.u/hamster), NDV-OSM (1 × 10^9^ iu/hamster) or saline (control), and were sacrificed 48 h after treatment for histopathological examination of tumors. Microphotographs show Hematoxylin/Eosin staining, magnification X100). The percentage of necrotic area in each group (*n* = 5) is represented in the right panel. * *p* < 0.05, ** *p* < 0.01

Orthotopic pancreatic cancer tumors were established in Syrian hamsters by inoculation of HaP-T1 cells. This model recapitulates the key genetic and histopathological characteristics of human PDAC [31, 34], forming highly aggressive and infiltrative adenocarcinomas as shown in Fig. 4a. Once tumor masses were evident by ultrasound or physical examination (average volume 200 mm^3^), hamsters were divided into groups that received different doses of either Ad-OSM or NDV-OSM, or were left untreated (injection of saline solution). A schematic representation of the experimental setting is shown in the right panel of Fig. 4a. Viruses were administered by direct intratumoral injection (day 0), and the same dose was repeated at day 3. This schedule was chosen based on previous experience showing that the transgene expression of repeated Ad administrations is blocked by neutralizing antibodies (NAb) as early as 7 days after the first virus inoculation (Additional file 1: Figure S2 and Additional file 2). Quantification of hOSM in serum of animals revealed important differences between viruses. Infection of Ad-OSM was very efficient inducing hOSM secretion, causing a transient, dose-dependent systemic elevation of the cytokine (Fig. 4b). The efficacy of the second virus administration was demonstrated in a separate group of hamsters that received 2.5 × 10^8^ iu Ad-OSM and were monitored for serum hOSM at days 1, 3 and 4 (Fig. 4c). Active virus replication was detected in these samples (Additional file 1: Figure S4). In the highest dose group (1 × 10^9^ iu/hamster), serum hOSM concentrations reached a peak of 3000 pg/ml at day 1, and caused severe toxicity with >50 % mortality during the first week after treatment. Equal doses of Ad-GFP or other replication-competent Ad carrying reporter genes were well tolerated (data not shown). Toxicity was not accompanied by elevation of transaminases in serum nor hematological abnormalities (data not shown), suggesting lung inflammation as the most likely cause of death [35]. In fact, histological examination of these organs revealed severe edema, inflammatory infiltrate and extracellular matrix accumulation (Additional file 1: Figure S3). In contrast, hamsters treated with 1 × 10^9^ or 5 × 10^9^ iu NDV-OSM showed no detectable hOSM in serum at any time. Only moderate concentrations of the cytokine (below 200 pg/ml) were observed when the virus was used at 1 × 10^10^ iu/hamster, but no treatment-related deaths occurred in this group. On the other hand, NDV-OSM was able to stimulate endogenous production of type I IFN in the animals starting at 1 × 10^9^ iu, whereas Ad caused no elevation of serum type I IFN even at the highest dose (Fig. 4b, right panel). All animals were sacrificed one month after initiation of treatment for direct assessment of tumor progression (Fig. 4d). The average tumor volumes were reduced in all treated groups as compared with untreated animals, but differences did not reach statistical significance. In the case of Ad-OSM, the intermediate dose (2.5 × 10^8^ iu) achieved tumor eradication in close to 50 % of the animals, but intensification of the treatment to improve the percentage of complete responses was not possible due to the toxicity associated with the release of hOSM into the circulation. In contrast, higher dose escalation of NDV-OSM was possible due to its lower toxicity, but no improvement in the therapeutic effect was observed by increasing the dose beyond 1 × 10^9^ iu/hamster. Additional hamsters treated with 2.5 × 10^8^ iu Ad-OSM or 1 × 10^9^ iu NDV-OSM were sacrificed 48 h after the first virus administration for histological analysis of tumor samples. The most significant finding was a marked increase in necrotic areas observed in tumors treated with NDV-OSM, accompanied by acute inflammatory infiltration (Fig. 4e). This is consistent with the strong oncolytic effect observed in PDAC cells infected with this virus in vitro.

### Sequential combination of Ad-OSM and NDV-OSM is safe and improves the antitumor effect of these agents

**Fig. 5:**
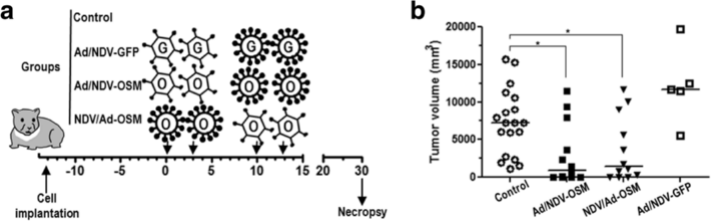
Sequential administration of low dose Ad-OSM and NDV-OSM is safe and inhibits progression of PDA tumors in hamsters. **a** Schematic representation of the experimental setting. Tumor-bearing hamsters were divided into 4 groups that received the following treatments: local administration of Ad-OSM at days 0 and 3, followed by two administrations of NDV-OSM at days 10 and 13 (Ad/NDV-OSM group, *n* = 12); the same viruses in the reverse order (NDV/Ad-OSM group, *n* = 12); Ad-GFP followed by NDV-GFP (Ad/NDV-GFP group, *n* = 5); or local injections of saline solution at days 0, 3, 10 and 13 (Control group, *n* = 19). All viral doses were 1 × 10^8^ iu/hamster. **b** Individual tumor volumes and group averages determined by necropsy one month after initiation of treatments. * *p* < 0.05

**Fig. 6:**
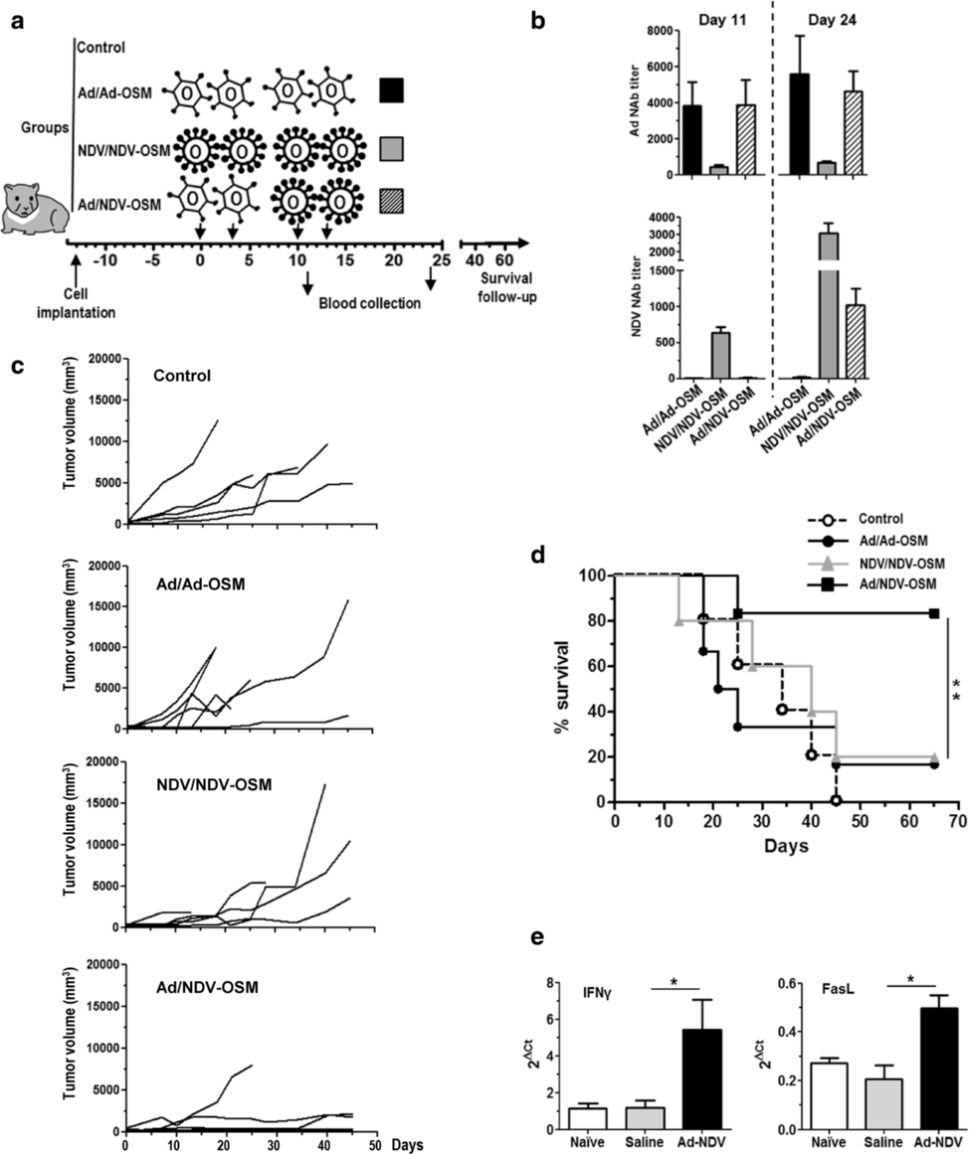
Sequential administration of Ad-OSM and NDV-OSM improves survival of tumor-bearing hamsters and stimulates immune response against cancer cells. **a** Schematic representation of the experimental setting. Tumor-bearing hamsters were divided into 4 groups (*n* = 6) that received the following treatments: Four local administrations of Ad-OSM (2.5 × 10^8^ iu/hamster) or NDV-OSM (1 × 10^9^ iu/hamster) at days 0, 3, 10 and 13 (Ad/Ad-OSM and NDV/NDV-OSM groups, respectively); or Ad-OSM (2.5 × 10^8^ iu/hamster) at days 0 and 3, followed by NDV-OSM (1 × 10^9^ iu/hamster) at days 10 and 13 (Ad/NDV-OSM group). **b** NAbs against Ad (top panel) or NDV (lower panel) were quantified in serum of hamsters at days 11 and 24. Ad/Ad-OSM, NDV/NDV-OSM and Ad/NDV-OSM groups are represented as black, grey and striped bars, respectively. **c** Progression of tumors in the different groups. Each line represents an individual hamster. **d** Percentage of surviving animals in each group. **e** Long-term survivors were sacrificed at day 70 and spleens were collected. Splenocytes were expanded for 5 days in the presence of irradiated HaP-T1 cells and IL-2, and then exposed for 8 h with fresh HaP-T1 cells. Expression of IFNγ and FasL was determined by qRT-PCR and compared with splenocytes from a group of untreated tumor-bearing hamsters sacrificed 40 days after cell implantation (saline), or healthy, naïve hamsters. * *p* < 0.05, ** *p* < 0.01

**Fig. 7:**
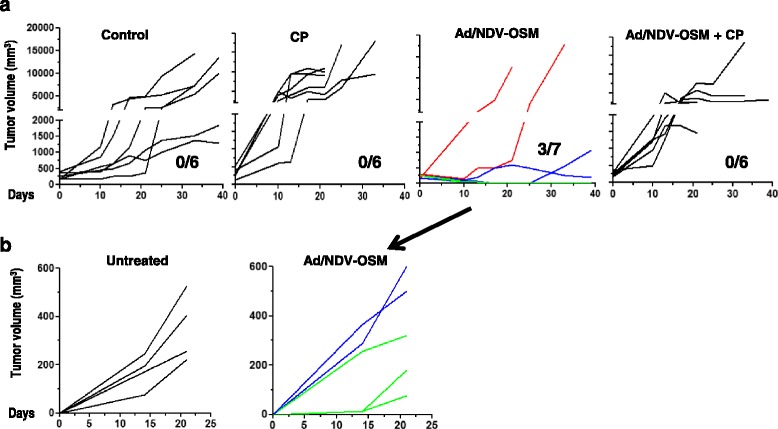
Immunosuppression abrogates the antitumor effect of OVs expressing OSM. The experimental setting is as described in Fig. 6. Treatment groups (*n* = 6/7) included control (saline solution injection); cyclophosphamide (CP); sequential administration of Ad-OSM and NDV-OSM (Ad/NDV-OSM) and the same treatment in combination with cyclophosphamide (Ad/NDV-OSM + CP). **a** Progression of tumors in the different groups. Each line represents an individual hamster. The numbers indicate the number of animals with complete response/total. In the Ad/NDV-OSM group the individuals with progressive disease, partial response and complete response are indicated by red, blue and green lines, respectively. **b** Long-term survivors from the Ad/NDV-OSM were subjected to a subcutaneous re-challenge with HaP-T1 cells. The graphs indicate the progression of tumors in each animal. A group of untreated hamsters bearing pancreatic tumors were used as a control

The results obtained in the first in vivo experiments (Fig. 4) with Ad-OSM and NDV-OSM indicated that safety and efficacy of this approach should be improved. To this end, we tested a new protocol in which low doses of the viruses (1 × 10^8^ iu) were combined sequentially in two cycles one week apart, as depicted in Fig. 5a. A combination of viruses expressing GFP was included to assess the role of direct viral oncolysis on the efficacy of treatment. As expected, no elevations of hOSM or IFNα were detected in the serum of treated hamsters in any of the groups (data not shown). Importantly, a significant antitumor effect was observed only in hamsters treated with the combinations of Ad-OSM and NDV-OSM, irrespective of the order of administration (Fig. 5b). This result suggests that the antitumor effect requires local OSM expression mediated by the OVs, which is effective at levels below the threshold required for appreciable secretion into the bloodstream. Since tolerance to this treatment was good and there was room for improving efficacy, we performed new experiments in which optimal intratumoral doses of Ad-OSM and NDV-OSM (2.5 × 10^8^ and 1 × 10^9^ iu, respectively) were used sequentially. Instead of tumor measurement after sacrifice, we studied long-term survival of animals. To assess the importance of combining different viruses, additional groups were included in which each cycle of treatment consisted of the same virus (Fig. 6a). This administration protocol allowed us to study the impact of humoral immunity on the efficacy of our virotherapy approach. When we examined the serum of animals in the middle of the second cycle of treatment (day 11), we confirmed the presence of NAbs at high titers against the virus used in the first cycle (Fig. 6b), suggesting that the only protocol that preserves the efficacy of the second round of infection is sequential administration. In agreement with this observation, this was the only group that showed a reduction in tumor progression (Fig. 6c) and a significant increase in survival compared with untreated animals in this aggressive tumor model (Fig. 6d). Finally, we studied if this protocol was able to stimulate the immune response against cancer cells. To this end, splenocytes obtained from long-term survivors were expanded in the presence of irradiated HaP-T1 cells and then exposed to the same cells. We observed an increase in IFNγ and FasL expression, compared with splenocytes obtained from tumor-bearing hamsters that received no treatment (control) or healthy (naïve) hamsters (Fig. 6e). This type of response has been previously associated with efficient antitumor immune responses in Syrian hamsters treated with OAVs expressing other cytokines [29]. To further explore the involvement of the immune system in the therapeutic effect of the treatment, we carried out an additional experiment in which one group of treated animals were immune suppressed by cyclophosphamide treatment, as previously described [36]. Although this drug enhances virus replication and spread in hamsters, the net balance was a negative impact on the antitumor effect. All animals treated with cyclophosphamide showed tumor progression, in contrast with 3 out of 7 hamsters with complete tumor remission and 70 % long-term survivors in the immune competent group (Fig. 7a). Finally, these animals received a subcutaneous re-challenge with HaP-T1 cells to determine the presence of a protective immune response. Only a slight retardation in tumor appearance was observed in 2 hamsters that had rejected their primary tumor (Fig. 7b). This result suggests that immune competence is required for the therapeutic effect of OVs expressing hOSM, although a robust immunological protection was not detected.

1

## Discussion

Progress in the field of virotherapy has led to a situation in which a wide repertoire of OVs is available for clinical testing, and the incorporation of immunostimulatory transgenes is showing the most promising results in patients [3–6, 37, 38]. New possibilities are now feasible, including the combination of OVs and the evaluation of different cytokines as therapeutic genes [39–42]. In this scenario the safety and efficacy of treatments not only depends on the potency and selectivity of virus replication, but also on other parameters such as the type of tumor and its immune properties, the expression of the transgene and the host antiviral responses. Our orthotopic PDAC model in Syrian hamsters has enabled us to characterize the properties of Ad and NDV OVs armed with hOSM, alone or in combination. One of the most relevant differences observed between both agents was their capacity to secrete hOSM into the circulation upon intratumoral administration. NDV-OSM required higher doses to cause elevations of this cytokine in the serum of animals as compared with Ad. This behavior was difficult to predict in vitro by analyzing the presence of hOSM in the conditioned medium of infected cells, probably because secreted proteins accumulate in the isolated compartment of the culture dish. We found no significant differences between either virus in these assays, but the situation in vivo revealed important differences. The stronger and faster cytolytic effect of NDV may determine that tumor cells initially infected with this virus can only express the transgene for a short period of time compared with Ad. Since virus amplification is rapidly controlled by the innate immune system [22, 43] and the secreted hOSM is constantly diluted in the bloodstream, the net balance is a low cytokine concentration in the circulation. The combination of a strong cytolytic effect and low capacity to secrete the therapeutic protein into the bloodstream is desirable when the transgene is potentially toxic, as is the case of OSM. In contrast, cells infected with Ad retain their viability for a prolonged period of time (48 to 72 h) and sustain a robust amplification of viral genomes, resulting in a very efficient expression of transgenes. As a consequence, Ad-OSM caused severe toxicity at relatively low viral doses (1 × 10^9^ iu/hamster), insufficient to exert a relevant cytolytic effect in the tumor mass. A similar observation has been recently reported with an oncolytic Ad expressing IL-12 [30]. Although we observed a clear dose-dependent production of hOSM in hamsters, establishment of the maximal tolerated dose in fully permissive hosts such as humans may be difficult because expression of the transgene will largely depend on the replication of viral genomes in tumors. Whereas the biological effect of hOSM may account for most of the antitumor effect observed with Ad-OSM, in the case of NDV-OSM the contribution of direct viral oncolysis may have certain relevance. In any case local expression of hOSM is crucial also for this virus, because no significant antitumor effect was observed when the NDV-GFP virus was used in this model, alone (data not shown) or in combination with Ad-GFP. Another peculiarity of NDV-OSM was its strong capacity to stimulate the endogenous production of type I IFN. Again, this was observed only in vivo, indicating that the source of type I IFN is the hamster immune system and not the tumor parenchyma. As type I IFN is essential in promoting CD8 mediated immune responses the concomitant expression of OSM and type I IFN may lead to synergistic immunostimulatory effects. Detailed analysis of the mechanism of action requires generation of new tools to study the immune system in Syrian hamsters, but our data demonstrate that local expression of OSM mediated by repeated administration of antigenically unrelated OV is needed to induce antitumor effects in a difficult-to treat- neoplasm. The therapeutic effect is associated with the induction of cellular responses against cancer cells and requires a competent immune system, although efficient protection from tumor recurrence at distant sites was not demonstrated in re-challenge experiments. This may reflect the importance of tumor microenvironment in the immune evasion of PDAC [44].

## Conclusions

Our study provides evidence that OSM promotes the expression of immunostimulatory genes in PDAC cells. Incorporation of this cytokine into oncolytic Ad and NDV viruses does not impair their replicative and cytolytic potential. Syrian hamsters carrying orthotopic PDAC tumors are sensitive to hOSM and permissive for replication of both OVs. Local expression of hOSM is required for the antitumor effect of Ad-OSM and NDV-OSM, but serum concentrations above 3000 pg/ml cause severe toxicity. Sequential administration of Ad-OSM and NDV-OSM avoids the interference of neutralizing antibodies, allowing two cycles of hOSM expression in tumors. This protocol achieves a significant antitumor effect and prolongs the survival of animals using safe doses of viruses. We believe this principle can increase the safety and efficacy of other immunovirotherapy approaches.

## Methods

### Cells

Syrian hamster cells HaP-T1 (DSMZ ACC 222) and H2T (courtesy of Dr. CM Townsend, University of Texas Medical Branch, TX, USA), and human cell lines HuH-7 (JCRB Genebank, Japan), Hep2 (ATCC CCL-23), A549 (ATCC CCL-185), BxPC-3 (ATCC CRL-1687) and PANC-1 (ATCC CRL-1469), were maintained in Dulbecco’s modified Eagle’s medium (Invitrogen) supplemented with 10 % fetal calf serum, 100 μg/ml streptomycin and 100 units/ml penicillin. For STAT3 activation analysis by western blot, 1.5 × 10^5^ HaP-T1 cells were seeded in a 6-well plate and 24 h later cells were treated with 20 ng/ml of hOSM (R&D) for 20 and 35 min. For quantification of IL-15Rα and ICAM1 mRNA levels, 1.2 × 10^4^ HaP-T1 or PANC-1 cells were seeded in a 96-well plate and 24 h later were treated with 20 ng/ml of hOSM for 24 and 48 h.

### Plasmid constructions

The coding sequence of hOSM was obtained as previously described [45]. The genome of Ad-OSM was based on the previously described virus Ad-IL12G [32], in which the single chain IL-12 coding sequence was replaced by hOSM, using standard molecular biology techniques. The same procedure was used to incorporate the HSV-1 thymidine kinase gene in the genome of the Ad-TK9 virus. The following specific primers: S_SacII_NDV_hOSM: CCGCGGTTAGAAAAAATACGGGTAGAACCGCCACCatgGGGGTACTGCTCACACAG and A_SacII_NDV_hOSM: CCGCGGATCATCTCCGGCTCCGGTTCGGGC were used to amplify the transgene in order to insert the hOSM sequence into the NDV genome between the P and M genes. The same strategy was used to clone GFP into the NDV genome using the following primers to amplify GFP by PCR as described for hOSM: S_SacII_NDV_GFP:CCGCGGTTAGAAAAAATACGGGTAGAACCGCCACCATGGTGAGCAAGGGCGAGG and A_SacII_NDV_GFP: CCGCGGATTACTTGTACAGCTCGTCC.

### NDV virus rescue

The NDV LaSota mutant viruses engineered to express eGFP or hOSM were generated as previously described [46] with some modifications. A549 and Hep2 cells were infected in a 6-well plate with MVA-T7 and one hour later, virus was removed and cells were transfected with viral genomes as described before [46]. Eighteen hours later, both cells and supernatants were mixed and injected into 8-day old chicken embryonated eggs. After three days of incubation, virus rescue was detected by the hemagglutination assay and sequenced by RT-PCR to verify insert fidelity. Immunofluorescence in Hep2 cells was performed to titrate rescued virus. To prepare virus stocks, 8-days old chicken embryonated eggs were used to grow original rescued virus by injecting approximately 500 iu per egg. Two days later, egg allantoic fluid was collected and virus stocks were prepared and frozen at −80 °C.

### NDV virus titration

NDV stocks were titrated by indirect immunofluorescence in Hep2 cells using polyclonal anti-NDV sera obtained from rabbits immunized with the virus followed by the use of a secondary goat anti-rabbit IgG (H + L) secondary antibody, Alexa Fluor® 488 conjugated (Life Technologies). Virus titer was determined by calculating TCID50 using the Reed-Muench method [47]**.**

### Adenovirus production and quantification

All adenoviral vectors were derived from human serotype 5. Plasmids containing viral genomes were linearized by PacI digestion (New England Biolabs), ethanol-precipitated and transfected in HEK-293 cells by Lipofectamine 2000 (Invitrogen). Cells were collected when the cytopathic effect was evident, and viral particles were obtained by 3 cycles of freeze/thaw. Viral clones were isolated by serial dilution in HEK-293 cells before amplification in the same type of cells.

Virus was purified by ultracentrifugation in a CsCl gradient. Infectious units were quantified using the Adeno-X rapid titer kit (Clontech). The Ad-WTLuc virus [29] is based on wild type adenovirus type 5 with insertion of the luciferase gene into the E3 region (substitution of the E3-6.7 K/gp19K genes). The same location was used to incorporate an EGFP expression cassette in the Ad-WT-CMV-GFP virus [30].

### Animals and treatment procedures

Orthotopic pancreatic cancer tumors were established in Syrian (Golden) hamsters (*Mesocricetus Auratus*; *HSD HAN: AURA,* 5 weeks of age, Harlan) using a method modified from Abraham et al. [34]. HaP-T1 cells (2 × 10^6^ cells resuspended in 50 μl saline) were injected into the splenic lobe of the pancreas through lateral laparotomy under inhaled anesthesia. This anatomical location facilitates repeated intratumor injection of treatments without the need of further laparotomies, resulting in a refinement of the animal procedure. When tumors reached at least 200 mm^3^ (typically 2 weeks after cell implantation), local administration of OAVs were performed by percutaneous injection in a total volume of 50 μl saline solution. Control animals received the same volume of vehicle. Average pre-treatment tumor volume was equivalent in all experimental groups. Tumor volumes were calculated at necropsy using the formula V = (Dxd^2^)/2, where D and d are the major and minor diameters, respectively. Re-challenge experiments were carried out by subcutaneous inoculation of 1 × 10^6^ HaP-T1 cells. Cyclophosphamide was administered intraperitoenally. The schedule consisted of an initial dose of 140 mg/Kg one week before the first virus administration, and then 100 mg/Kg twice weekly for 2 weeks [36]. All procedures were carried out following protocols approved by the local ethics committee in accordance with recommendations for proper care and use of laboratory animals.

### Histological analysis and quantification of tumor necrosis

Tumor sections were fixed, paraffin-embedded and stained with Hematoxylin and Eosin following standard procedures. Samples were randomized (Research randomizer, https://www.randomizer.org/) and the percentage of necrotic area in tumors was quantified using Image J software in a blinded fashion.

### Western blot assays

Western blot analyses were performed as previously described [17].

### Analysis of gene expression

Total RNA from cells was extracted using the automated MagMax Express 96 system (Applied Biosystems) using the Magmax-96 total RNA isolation kit (Life Technologies). Reverse transcription (RT) was performed as previously reported [48]. Real-time polymerase chain reactions (PCRs) were performed with iQ SYBR Green supermix (Bio-Rad) in a CFX96 system from Bio-Rad, using specific primers for each gene (Table 1). The amount of each transcript was expressed by the formula: 2^ct(β-actin or CD3)−ct(gene)^, ct being the point at which the fluorescence rises appreciably above the background levels.

### Quantification of human OSM protein levels

Concentration of hOSM in cell supernatants was determined 24 h after infection with Ad-OSM or NDV-OSM by enzyme-linked immunosorbent assay (ELISA, R&D) according to the manufacturer’s instructions. The same method was used to measure hOSM in the serum of hamsters.

**Table 1:** Primers used in this study

Gene	Sense primer (5´-3´)	Antisense primer (5´-3´)
Hamster IL-15Rα	ACACAAATACTGCCCACTGG	TCCAAGGTCATTGTTGCTGC
Hamster ICAM1	CATGGAGCCAGTTTCTCATG	ATCACTTTCTGCATGGTGCC
Human IL-15Rα	GGAACCACAGAGATAAGCAG	CCTTGACTTGAGGTAGCATG
Human ICAM1	CCGAGCTCAAGTGTCTAAAG	CCTTTTTGGGCCTGTTGTAG
Hamster IFNγ	GGCCATCCAGAGGAGCATAG	CCATGCTGCTGTTGAAGAAGTTAG
Hamster FasL	AAGAAGAGGAAGGACCACAG	TTTTCAGAGGGTTGACTCGG
Hamster CD3	CGGCGAAAGTATGGTTTACC	TGCTCGTTCTTCAACAGAGC
Hamster Tap1	CGTTCTCAGTTATGTAGCAG	CCCGTGAAGAAAGGAATGGC
Hamster B2M	ATGCCATCCAGCGTCCCC	ATCGGTCGCAGTGGGTGTAA
Hamster OAS	CTCATCCGCCTGGTCAAGC	GGGTCCAGGATCACAGGC
Hamster GBP1	TGGCATCAGAGATCCACATG	ACACACCACATCCAGATTCC
Hamster actin	GTCGTACCACTGGCATTGTG	GTCACGCACAATTTCCCTCT

### Analysis of immune response against HaP-T1 cells

Spleens were removed from the euthanized animals, and splenocytes were isolated by passing the spleens through 70 μm nylon mesh filters. Splenocytes (3 × 10^6^ cells/well) were co-cultured with 1.5 × 10^5^ irradiated HaP-T1 cells in 24-well plates for 8 days. On day 3, 50 units/ml of IL-2 were added to co-cultures. After 8 days, splenocytes were collected and seeded (1 × 10^5^ cells/well) together with 1 × 10^4^ fresh HaP-T1 cells in 96-well plates. After 8 h of co-culture, the cells were collected in lysis/binding solution (Life Technologies) for total RNA extraction and subsequent IFNγ and Fas Ligand mRNA expression study. The amount of each transcript was expressed by the formula: 2^ct(CD3)−ct(gene)^.

### Type I IFN bioassay

The antiviral activity of hamster type I IFN present in serum or cell supernatants was analyzed by measuring its ability to protect hamster H2T cells against the cytopathic effect of encephalomyocarditis virus (EMCV). The assay was performed in a 96-well microtiter plate. First, 2 × 10^4^ H2T cells per well were seeded in 150 μl of medium containing serial dilutions of serum or conditioned media from cells infected with Ad or NDV. The viruses were previously UV-inactivated. After incubation for 24 h, cells were infected with EMCV (5 × 10^6^ pfu per well), and 24 h later, the cytopathic effect was measured by staining with crystal violet dye solution (0.5 % in 1/4 v/v methanol/water). The optical density was read at 595 nm. At the same time, serial dilutions of human IFNα (PBL Biomedical Laboratories) were tested to obtain a standard curve. Results are expressed as units/ml and were calculated interpolating the optical density of each sample in the standard curve.

### Quantification of neutralizing antibodies

Anti-adenovirus type 5 neutralizing antibodies were determined using a modified luciferase-based virus neutralization assay, as previously described [49]. In the case of NDV, the reporter virus NDV-GFP was serially diluted in a 1:2 series. A virus dilution corresponding to the last dose presenting close to 100 % GFP signal in Hep2 cells was later used for incubation at 25 °C for 2 h with hamster sera. Ab neutralizing titers were obtained in quadruplicate using the Reed-Muench method to calculate the inhibitory ED50 also in Hep2 cells.

### Statistical analysis

The Mann Whitney test was applied for statistical comparison of two groups. Comparisons of more than two groups were performed by ANOVA with Bonferroni correction. Survival differences were analyzed by the Log-Rank (Mantel-Cox) test using the GraphPad Prism program (GraphPad Software). Unless otherwise stated, triplicate samples were used for in vitro experiments. For animal studies, a sample size estimate was obtained from http://www.biomath.info/power/index.htm. The number of animals per group is indicated in the scatter plot graphs. Unless otherwise stated, all experiments were repeated at least twice.

## Additional files

Additional file 1:
**Additional Figure S1 Measurement of type I IFN in conditioned media from HaP-T1 cells infected with of Ad or NDV.** H2T cells were exposed for 8 h to conditioned media from HaP-T1 cells infected during 24 h with the indicated viruses. mRNA levels of the type I IFN stimulated genes OAS (a) and GBP1 (b) were determined by qRT-PCR. H2T cells treated with conditioned media from uninfected cells (control) or cells treated with 500 units/ml recombinant human IFNα are included as negative and positive controls, respectively (representative results of at least 2 experiments performed in triplicate). *** *p* < 0.001. **Additional Figure S2 Rapid onset of NAbs reduces the efficacy of Ad redosing.** Hamsters bearing orthotopic pancreatic cancer tumors received a local administration of Ad-WT-Luc (1 × 10^9^ iu/hamster) at day 0 and were then separated into two groups (*n* = 4). One of them received a second administration of the same dose of virus at day 3 (a) and the other one received it at day 7 (b). Expression of luciferase was quantified by in vivo bioluminescence imaging at the indicated times. The graphs show total light emission (in photons/s) measured from the tumor area. (c) Representative image of a hamster expressing luciferase at day 1, showing the region of interest for quantification. Note the predominant light emission in the anatomical region corresponding to the injected pancreatic tumor (left panel, ventral view; right panel, lateral view). In the artificial color code, red and blue represent maximal and minimal light emissions, respectively. **Additional Figure S3 Elevation of OSM in serum causes severe lung inflammation in Syrian hamsters.** Animals treated with 1 × 10^9^ iu Ad-OSM show signs of toxicity and most of them die or require euthanasia during the first week after virus administration. The microphotographs show histopathological examination of lungs from representative animals from the untreated (control) and Ad-OSM-treated groups (Hematoxylin-Eosin, x200). Note the presence of edema, inflammatory infiltrate and deposition of extracellular matrix. **Additional Figure S4 Ad-OSM replicates in PDAC tumors in vivo.** Hamsters bearing orthotopic pancreatic cancer tumors received a local administration of Ad-OSM at 2.5 × 10^8^ iu/hamster (*n* = 5) or a first-generation adenoviral vector encoding luciferase (AdCMVLuc) at 2 × 10^9^ iu. Hamsters were sacrificed 4 days later for tumor collection. (a) Quantification of infectious units in tumor lysates (expressed as iu/mg tissue). (b) Detection of hexon expression in tumor cells by immunohistochemistry. A cluster of positive cells is marked by a black arrow (200x magnification). (PDF 1298 kb) Additional file 2:
**Additional methods.** In vivo bioluminescence detection. Anesthetized hamsters received an intraperitoneal injection of 9 mg D-Luciferin Firefly (Sigma) dissolved in 300 μl PBS. Five minutes later they were placed in a dark chamber connected to an in vivo luminescent detection system (IVIS, Xenogen). Photon emission was quantified using the Living Image Software (Xenogen). (DOC 22 kb)

## Abbreviations

Ad: Adenovirus
hOSM: Human oncostatin
IFN: Interferon
IL: Interleukin
iu: Infectious units
LIF: Leukemia inhibitory factor
NA b: Neutralizing antibodies
NDV: Newcastle disease virus
OV: Oncolytic virus
PDAC: Pancreatic ductal adenocarcinoma
