# Supplementary figures and images for: Enhanced therapeutic effect using sequential administration of antigenically distinct oncolytic viruses expressing oncostatin M in a Syrian hamster orthotopic pancreatic cancer model
Source: Mol Cancer. 2015 Dec 16;14:210. doi: 10.1186/s12943-015-0479-x (PMC4681018; doi:10.1186/s12943-015-0479-x)

## Additional figure 1

**a**

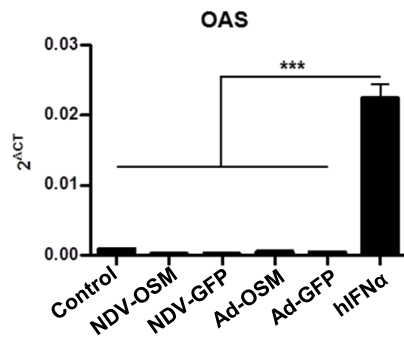

**b**

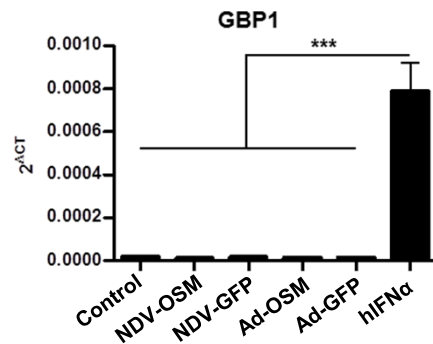

Additional figure 2

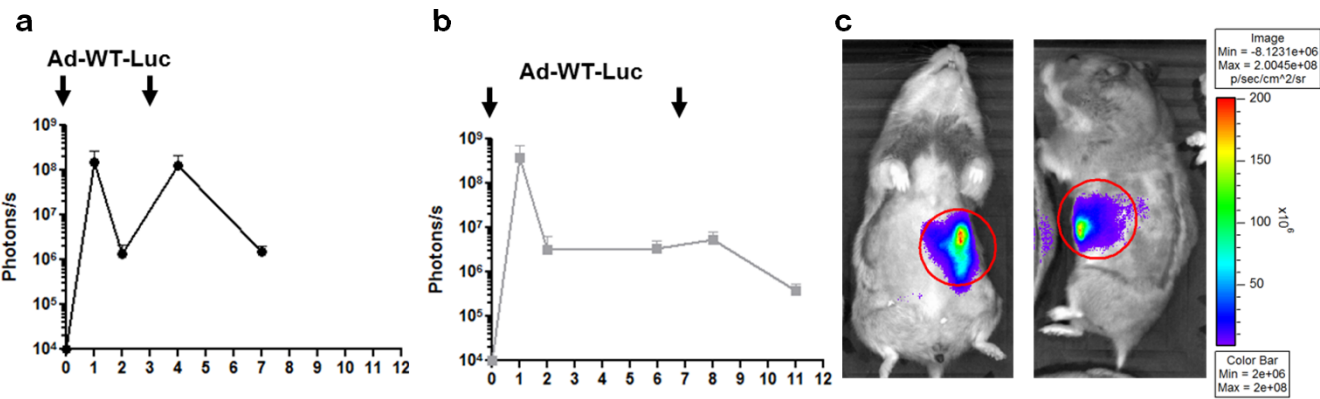

### Additional figure 3

**Control**

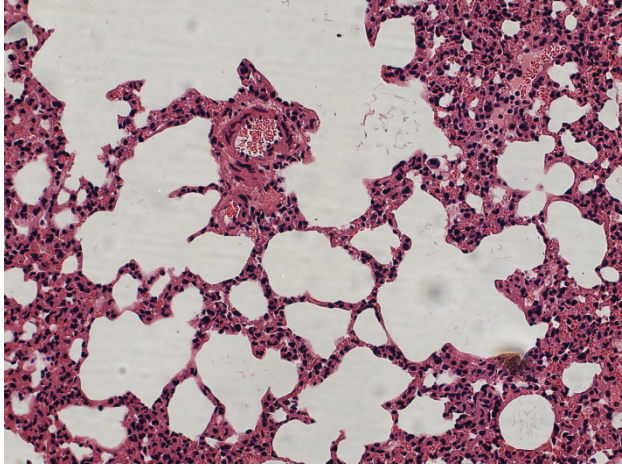

**Ad-OSM**

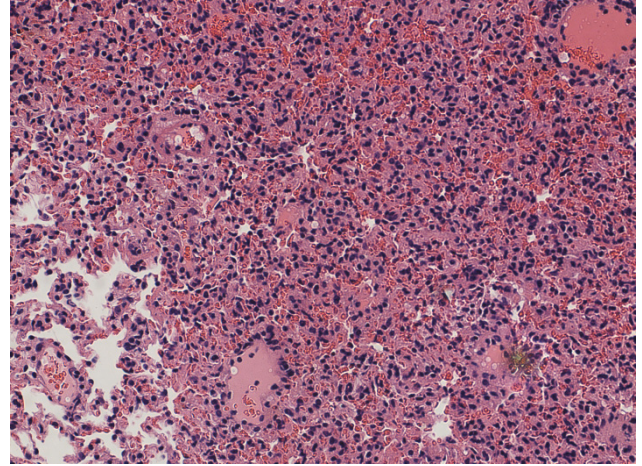

Additional figure 4

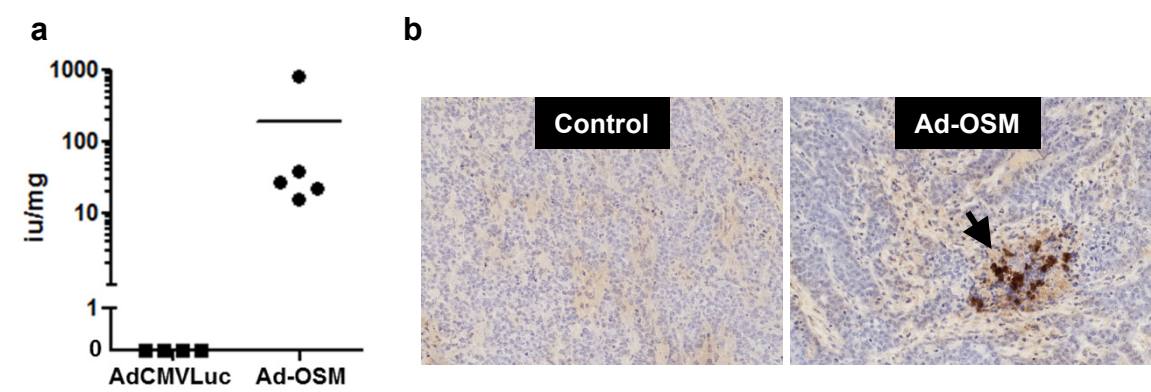

Supplement: Additional file 1: — Additional Figure S1 Measurement of type I IFN in conditioned media from HaP-T1 cells infected with of Ad or NDV. H2T cells were exposed for 8 h to conditioned media from HaP-T1 cells infected during 24 h with the indicated viruses. mRNA levels of the type I IFN stimulated genes OAS (a) and GBP1 (b) were determined by qRT-PCR. H2T cells treated with conditioned media from uninfected cells (control) or cells treated with 500 units/ml recombinant human IFNα are included as negative and positive controls, respectively (representative results of at least 2 experiments performed in triplicate). *** p < 0.001. Additional Figure S2 Rapid onset of NAbs reduces the efficacy of Ad redosing. Hamsters bearing orthotopic pancreatic cancer tumors received a local administration of Ad-WT-Luc (1 × 10^9^ iu/hamster) at day 0 and were then separated into two groups (n = 4). One of them received a second administration of the same dose of virus at day 3 (a) and the other one received it at day 7 (b). Expression of luciferase was quantified by in vivo bioluminescence imaging at the indicated times. The graphs show total light emission (in photons/s) measured from the tumor area. (c) Representative image of a hamster expressing luciferase at day 1, showing the region of interest for quantification. Note the predominant light emission in the anatomical region corresponding to the injected pancreatic tumor (left panel, ventral view; right panel, lateral view). In the artificial color code, red and blue represent maximal and minimal light emissions, respectively. Additional Figure S3 Elevation of OSM in serum causes severe lung inflammation in Syrian hamsters. Animals treated with 1 × 10^9^ iu Ad-OSM show signs of toxicity and most of them die or require euthanasia during the first week after virus administration. The microphotographs show histopathological examination of lungs from representative animals from the untreated (control) and Ad-OSM-treated groups (Hematoxylin-Eosin, x200). Note th [file 12943_2015_479_MOESM1_ESM.pdf]
