# Supplementary material for: Enhanced therapeutic effect using sequential administration of antigenically distinct oncolytic viruses expressing oncostatin M in a Syrian hamster orthotopic pancreatic cancer model
Source: Mol Cancer. 2015 Dec 16;14:210. doi: 10.1186/s12943-015-0479-x (PMC4681018; doi:10.1186/s12943-015-0479-x)
Supplement: Additional file 2: — Additional methods. In vivo bioluminescence detection. Anesthetized hamsters received an intraperitoneal injection of 9 mg D-Luciferin Firefly (Sigma) dissolved in 300 μl PBS. Five minutes later they were placed in a dark chamber connected to an in vivo luminescent detection system (IVIS, Xenogen). Photon emission was quantified using the Living Image Software (Xenogen). (DOC 22 kb) [file 12943_2015_479_MOESM2_ESM.doc]

**Additional methods**

**In vivo bioluminescence detection.**

Anesthetized hamsters received an intraperitoneal injection of 9 mg D-Luciferin Firefly (Sigma) dissolved in 300 μl PBS. Five minutes later they were placed in a dark chamber connected to an *in vivo* luminescent detection system (IVIS, Xenogen). Photon emission was quantified using the Living Image Software (Xenogen).
